# Invasive neuroendocrine carcinoma of the breast: a population-based study from the surveillance, epidemiology and end results (SEER) database

**DOI:** 10.1186/1471-2407-14-147

**Published:** 2014-03-04

**Authors:** Jun Wang, Bing Wei, Constance T Albarracin, Jianhua Hu, Susan C Abraham, Yun Wu

**Affiliations:** 1 Department of Oncology, General Hospital, Jinan Command of the People’s Liberation Army, Jinan, China; 2 Department of Pathology, West China Hospital, Sichuan University, Chengdu, China; 3 Department of Pathology, The University of Texas MD Anderson Cancer Center, 1515 Holcombe Blvd, Houston, TX 77030, USA; 4 Department of Biostatistics, The University of Texas MD Anderson Cancer Center, Houston, TX, USA

**Keywords:** Neuroendocrine carcinoma, Endocrine carcinoma, Invasive carcinoma, Breast, SEER registry

## Abstract

**Background:**

Neuroendocrine carcinoma (NEC) of the breast is a rare type of carcinoma that has not been well studied or characterized. Of the limited number of studies reported in the literature, most are case reports. A few small retrospective series studies have been reported.

**Methods:**

We reviewed data on 142 cases of mammary NEC recorded in the surveillance, epidemiology, and end results (SEER) database during 2003–2009 and evaluated disease incidence and patient age, sex, and race/ethnicity; clinicopathologic characteristics; and survival in comparison to invasive mammary carcinoma, not otherwise specified. We also performed univariate and multivariate analyses to identify prognostic factors in this disease.

**Results:**

Review of the 142 SEER cases revealed that NEC is an aggressive variant of invasive mammary carcinoma. It generally occurred in older women (>60 years); present with larger tumor size (>20 mm), higher histologic grade, and higher clinical stage; and result in shorter overall survival and disease-specific survival than invasive mammary carcinoma, not otherwise specified (IMC-NOS). Overall survival and disease-specific survival were shorter in NEC at each stage than in IMC-NOS of the same stage. Furthermore, when all NEC and IMC-NOS cases were pooled together, neuroendocrine differentiation itself was an adverse prognostic factor independent of other known prognostic factors, including age, tumor size, nodal status, histologic grade, estrogen/progesterone receptor status, and therapy.

**Conclusions:**

NEC is a rare but aggressive type of mammary carcinoma. Novel therapeutic approaches should be explored for this uniquely clinical entity.

## Background

Neuroendocrine carcinoma (NEC) of the breast is a very rare malignant tumor. Only a limited number of studies on NEC have been reported in the literature, most of them anecdotal case reports. Very few are series studies [1-11]. Much of the current limited knowledge of this disease is based on these small retrospective series and thus is subject to selection/referral bias. Therefore, very little is known about the disease incidence, age and sex predilection, race/ethnicity distribution, clinicopathologic characteristics, and survival.

To gain more insight into mammary NEC, we took advantage of a large database of cancer cases collected during the last two decades from surveillance, Epidemiology, and end results (SEER) registries. Using SEER data, we evaluated the incidence and clinical course of mammary NEC in comparison to its more common counterpart, invasive mammary carcinoma, not otherwise specified (IMC-NOS).

## Methods

### Data acquisition and patient selection

We utilized SEER data released in April 2012 [12]. The SEER database includes data from 9 population-based registries (1990–1999) and 18 population-based registries (2000–2009) which cover approximately 26% of U.S. cancer patients. The SEER database classifies cancer histology and topography information on the basis of the third edition of the International Classifications of Diseases for Oncology (ICD-O-3). We included all cases of invasive carcinoma (behavior code/3) of the breast (C500-509) and the study cohort of mammary NEC (8013/3 and 8246/3). No mammary NEC cases were identified in the SEER database before 1998. Of note, mammary NEC was strictly defined for the first time in 2003 by the World Health Organization (WHO) as >50% of the tumor cells expressing neuroendocrine markers [13]. We, therefore, focused our study on cases diagnosed from 2003 to 2009. Patients with stage I-IV invasive mammary carcinoma diagnosed between 2003 and 2009 were identified from the SEER database (n = 381,644) to compare with the NEC cohort (n = 142). We also performed survival analyses on 72 cases of mammary NEC and 382,453 control cases of IMC, NOS identified from the SEER database based on the same ICD codes between 1998 and 2002.

### Data analysis

Descriptive statistics were calculated for demographic and clinicopathologic factors, and differences in these between the NEC and IMC-NOS cohorts were evaluated using the chi-square or Fisher exact test, as appropriate. Age and tumor size were analyzed as continuous variables, and statistical differences in the mean values were assessed using the Student *t*-test. Rates of disease-specific survival (DSS) and overall survival (OS) were used as primary endpoints. Survival was measured from the date of diagnosis to the date of death, the date last known to be alive, or November 30, 2009. To determine the effects of different variables on OS and DSS, we performed a univariate survival analysis using the Kaplan-Meier method, and the significance was assessed using the log-rank test. A multivariate analysis was performed using the Cox proportional hazards model. The estimated risks for OS or DSS were calculated as hazard ratios (HRs) with 95% confidence intervals (CIs).

All tests were 2-tailed, and a *P-*value <0.05 was considered statistically significant. Statistical analyses were performed using STATA software version 12.0 (Stata Corporation, College Station, TX).

## Results

### Incidence

During the period from 2003 to 2009, a total of 381,644 cases of invasive mammary carcinoma were registered in the SEER database; in comparison, only 142 cases of invasive NEC were registered, which comprised <0.1% of total invasive carcinomas of the breast, much less than the 2-5% rate reported by the World Health Organization [13].

Using the rate session in the SEER *Stat software (version 7.1.0; Surveillance Research Program, NCI, Bethesda, MD), we calculated age-adjusted incidence rates for NEC of the breast as 0.23 per 1 million-years in all populations (95% CI: 0.18-0.29), 0.41 per 1 million-years in the female population (95% CI: 0.31-0.53), and 0.01 per 1 million-years in the male population (95% CI: 0.00-0.06).

### Clinicopathologic characteristics

The clinicopathologic characteristics of the 142 NEC patients were compared with those of IMC-NOS, and the results are summarized in Table 1.

**Table 1:** Baseline demographic and clinicopathologic features of the mammary NEC cohort and the invasive mammary carcinoma control cohort from the SEER database (2003–2009)

**Characteristics**	**NEC**		**Invasive mammary carcinoma**		***P***
Age, years, mean ± SD	63.6 ± 14.9		61.3 ± 14.2		0.029
Tumor size (mm), mean ± SD	31.9 ± 31.1		22.5 ± 24.1		<0.0001
	**No. of patients**	**%**	**No. of patients**	**%**	
Sex					0.06^a^
Male	3	2.1	2,909	0.8	
Female	139	97.9	378,735	99.2	
Race					NS
White	121	85.2	312,513	81.9	
Black	13	9.2	38,975	10.2	
Other	8	5.6	30,156	7.9	
AJCC TNM stage					<0.0001*
I	32	22.5	173,349	45.4	
II	52	36.6	125,129	32.8	
III	16	11.3	42,020	11.0	
IV	34	23.9	18,844	4.9	
Unknown	8	5.6	22,302	5.8	
Regional lymph node					0.05*
Negative	52	36.6	214,745	56.3	
Positive	40	28.2	109,741	28.8	
Unknown	50	35.2	57,158	14.9	
Grade					<0.0001*
I	17	12.0	75,043	19.7	
II	30	21.1	147,540	38.7	
III	60	42.3	126,919	33.3	
Unknown	35	24.7	32,142	8.3	
ER status					0.003**
Negative	37	26.1	74,093	19.4	
Borderline	0	0	812	0.2	
Positive	77	54.2	274,474	71.9	
Unknown	28	19.7	32,265	8.5	
PR status					<0.0001**
Negative	59	41.6	114,069	29.9	
Borderline	0	0	2,626	0.7	
Positive	53	37.3	228,877	60.0	
Unknown	30	21.1	36,214	9.5	
Surgery					<.0001*
No	33	23.2	28,888	7.6	
Yes	109	76.8	352,865	92.4	
Radiation					0.038
No	91	64.1	211,458	55.4	
Yes	51	35.9	170,186	44.6	

NEC, neuroendocrine carcinoma; TNM, tumor-lymph nodes-metastasis; LN, lymph node; ER, estrogen receptor; PR, progesterone receptor; NS, not significant.

^a^Fisher exact test.

*Cases with other or unknown status were excluded from statistical analysis.

**Cases with borderline or unknown status were excluded from statistical analysis.

#### Age, sex, and ethnicity

The mean age at diagnosis of patients with NEC was 64 years (range 26–99 years; median 63 years). NEC patients were significantly older (*P* = 0.029) than those with IMC-NOS (range 10–114 years; mean 61 years; median 61 years).

The distribution of ethnicity in cases of NEC of the breast was similar to that in cases of IMC-NOS (Table 1). There were proportionally more males with NEC than with IMC-NOS (2.1% vs. 0.8%) but is not statistically significant (*P* = 0.06) (Table 1).

#### Stage at diagnosis

##### Tumor size (T stage)

At diagnosis, NEC tumors were significantly larger than IMC-NOS tumors (*P* < 0.0001) (Table 1). The mean NEC size was 32 mm, whereas the mean IMC-NOS size was 23 mm.

##### Regional lymph node metastasis (N stage)

More patients in the NEC group than in the IMC-NOS group had positive regional lymph nodes at the time of diagnosis (borderline significant, *P* = 0.05) (Table 1). Excluding cases whose lymph node status was unknown, 43% of NEC cases and 34% of IMC-NOS cases presented with lymph node metastasis at the time of diagnosis.

##### TNM stage

The NEC cases presented with a higher TNM stage than the IMC-NOS cases (*P* < 0.0001) (Table 1). There were more patients with stage II-IV disease in the NEC group than in the IMC-NOS group. Whereas most of the IMC-NOS group presented with stage I disease, NEC patients most often presented with stage II disease, indicating either large tumor size or regional lymph node metastasis at the time of diagnosis.

#### Tumor grade

The tumors of the NEC group were of significantly higher histologic grade than those of the IMC-NOS group (*P* < 0.0001) (Table 1). Most of NEC tumors were grade III, whereas most of IMC-NOS tumors were grade II.

#### Receptor status

Most NECs of the breast were ER and PR positive. However, fewer NECs were ER and/or PR positive (67.9%) than IMC-NOS (79.7%) (Table 1). HER2 status is not available from the SEER database.

### Survival

**Figure 1:**
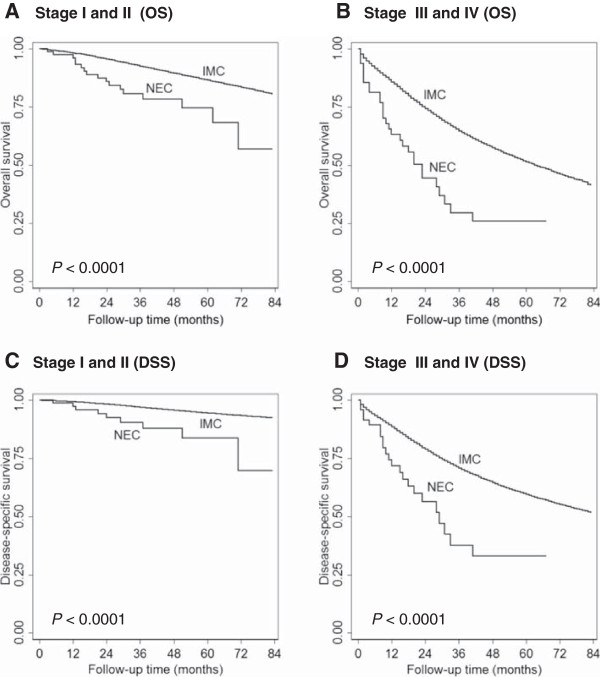
**Overall survival (OS) and disease-specific survival (DSS) comparisons between neuroendocrine carcinoma (NEC) and invasive mammary carcinoma, not otherwise specified (IMC) diagnosed between 2003 and 2009.** OS and DSS were significantly shorter in NEC than in IMC-NOS in both early stage disease **(A and C)** and advanced stage disease **(B and D)**.

The median survival of patients with NEC was 26 months (interquartile range [IQR], 12–48 months), which was much shorter than that of patients with IMC-NOS (median, 34 months; IQR, 16–56 months). The 5-year OS rates were also much lower in the NEC group than in the IMC-NOS group (*P* < 0.0001) (Table 2). As expected, the more advanced the disease stage at the time of presentation, the worse the clinical outcome. Therefore, we stratified patients by stage, showing that patients with stage I, II or III disease in the NEC group had lower OS rate than patients in the IMC-NOS group with the same stage disease (Table 2). In addition, survival analyses showed worse OS and DSS in stage I-II NEC than that in IMC, NOS patients with the same stage (Figure 1A, 1C). Similar results were seen for advanced stage NEC in comparison with IMC, NOS (Figure 1B, 1D).

**Table 2:** Overall survival in NEC cohort and invasive mammary carcinoma cohort according to clinical stage (2003–2009)

**Cohort**	***n***	**Median survival in months (IQR)**	**5-year OS rate (95% CI)**	***P***
**All**				<0.0001
NEC	135	26 (12–48)	53.6 (42.2-63.7)	
IMC	374,598	34 (16–56)	79.8 (79.6-79.9)	
**Stage I**				0.002
NEC	32	33 (17–51)	74.4 (43.4-90.0)	
IMC	170,778	36 (18–58)	89.6 (89.2-89.6)	
**Stage II**				<0.0001
NEC	49	30 (19–52)	73.9 (56.3-85.3)	
IMC	123,430	36 (17–58)	82.4 (82.1-82.7)	
**Stage III**				0.014
NEC	16	19 (13–41)	58.2 (21.0-82.8)	
IMC	41,422	29 (14–48)	72.4 (71.8-73.1)	
**Stage IV**				NS
NEC	32	12 (4–25)	20.7 (5.70-42.1)	
IMC	17,830	15 (5–30)	27.9 (26.9-29.0)	

Data from the Surveillance, Epidemiology and End Results Program, 2003 to 2009. NEC, neuroendocrine carcinoma; IMC, invasive mammary carcinoma; IQR, interquartile range; OS, overall survival; *n*, number of cases; HR, hazard ratio; CI, confidence interval; NS, not significant.

### Prognostic factors

**Figure 2:**
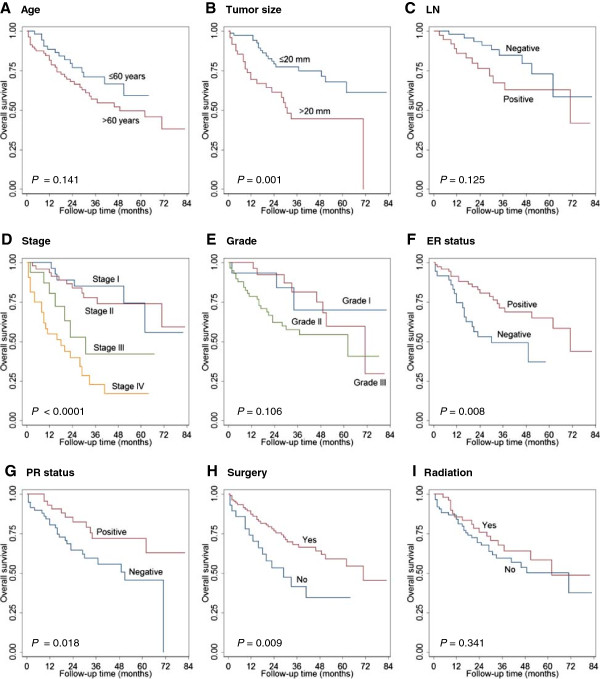
**Factors affecting overall survival (OS) of mammary NEC.** Age **(A)**, tumor size **(B)**, lymph node status **(C)**, stage **(D)**, histologic grade **(E)**, estrogen receptor (ER) and progesterone receptor (PR) status **(F, G)**, surgical resection **(H)** and radiation therapy **(I)** were analyzed.

**Figure 3:**
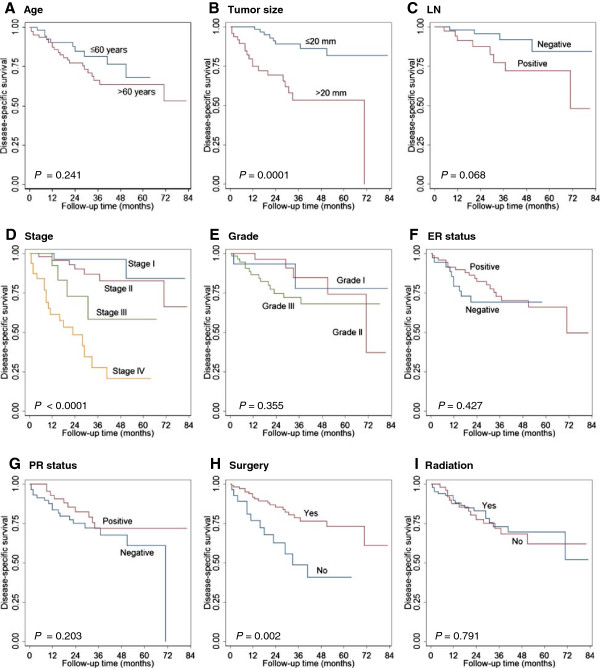
**Factors affecting disease-specific survival (DSS) of mammary NEC.** Age **(A)**, tumor size **(B)**, lymph node status **(C)**, stage **(D)**, histologic grade **(E)**, estrogen receptor (ER) and progesterone receptor (PR) status **(F, G)**, surgical resection **(H)** and radiation therapy **(I)** were analyzed.

Univariate analysis by the Kaplan-Meier method showed that larger tumor size (>20 mm), higher tumor stage, negative ER/PR status, and lack of surgical treatment were associated with shorter OS in the NEC cohort (Figure 2, Table 3). Older age (>60 years), larger tumor size (>20 mm), higher tumor stage, and lack of surgical treatment were associated with shorter DSS in the NEC cohort (Figure 3, Table 3). In multivariate analysis, only older age and positive lymph node status were independently prognostic for poor OS (*P* = 0.012 and *P* < 0.0001, respectively). Negative PR status, positive lymph node status and lack of surgery treatment were the only independent prognostic factor for DSS (*P* = 0.006, *P* < 0.0001 and *P* = 0.041) (Table 4).

**Table 3:** Univariate survival analysis (Kaplan-Meier) in selected subgroups of patients with NEC of the breast according to characteristics

**Characteristics**	**5-year DSS**				**5-year OS**		
	***n***	**Survival rate (95% CI)**	**HR (95% CI)**	***P***	**Survival rate (95% CI)**	**HR (95% CI)**	***P***
Sex				NS			NS
Male	2	Not reached			Not reached		
Female	133	66.2 (54.2-75.7)	Not reached		54.1 (42.7-64.3)	0.39 (0.05-2.84)	
Age (year)				NS			NS
≤60	55	67.9 (43.0-83.8)			59.1 (37.6-75.4)		
>60	80	63.7 (49.2-75.1)	1.56 (0.74-3.32)		49.5 (35.8-61.8)	1.58 (0.85-2.91)	
Race				0.031			NS
White	114	68.9 (55.4-78.9)			55.4 (42.8-66.4)		
Black	13	63.3 (28.6-84.6)	1.63 (0.56-4.70)		58.0 (26.1-80.2)	1.27 (0.50-3.25)	
Other	8	Not reached	3.15 (1.08-9.19)		Not reached	2.39 (0.94-6.11)	
AJCC TNM stage				<0.0001			<0.0001
I	32	84.3 (43.2-96.6)			74.4 (43.4-90.0)		
II	49	82.6 (64.4-92.0)	2.14 (0.44-10.4)		73.9 (56.3-85.3)	1.15 (0.42-3.14)	
III	16	58.2 (21.0-82.8)	5.75 (1.05-31.5)		42.2 (14.3-68.2)	3.26 (1.09-9.75)	
IV	32	20.7 (5.70-42.1)	18.0 (4.14-78.6)		17.1 (4.72-35.9)	6.92 (2.78-17.2)	
LN				NS			NS
Negative	52	84.2 (58.1-94.7)			72.8 (49.6-86.7)		
Positive	38	72.1 (48.8-86.1)	2.93(0.87-9.81)		62.7 (41.8-77.9)	1.96 (0.82-4.68)	
Size (mm)				<0.0001			0.002
≤20	73	81.8 (65.1-90.9)			67.9 (52.1-79.5)		
>20	48	53.4 (34.7-68.9)	4.82 (2.08-11.2)		44.4 (27.7-59.9)	2.70 (1.45-5.03)	
Grade				NS			NS
I	15	77.8 (31.6-94.7)			70.0 (29.9-90.0)		
II	29	74.2 (41.3-90.4)	1.12 (0.22-5.78)		59.6 (31.6-79.3)	1.18 (0.31-4.46)	
III	58	68.0 (51.0-80.2)	2.09 (0.47-9.28)		54.3 (38.9-67.3)	2.39 (0.72-7.98)	
ER status				NS			0.01
Negative	36	Not reached			Not reached		
Positive	72	66.0 (49.7-78.1)	0.85 (0.58-1.26)		64.8 (48.7-77.0)	0.65 (0.47-0.90)	
PR status				NS			0.022
Negative	58	60.8 (40.3-76.2)			45.6 (27.8-61.7)		
Positive	48	71.8 (53.3-84.0)	0.78 (0.52-1.15)		71.8 (53.3-84.0)	0.66 (0.46-0.94)	
Radiation				NS			NS
No	85	69.5 (54.1-80.6)			50.2 (35.4-63.2)		
Yes	50	62.0 (41.8-76.9)	1.10 (0.55-2.21)		58.3 (39.0-73.4)	0.75 (0.42-1.36)	
Surgery				0.003			0.012
No	28	40.6 (17.6-62.6)			34.6 (14.9-55.3)		
Yes	107	73.2 (59.7-82.8)	0.34 (0.16-0.69)		58.9 (45.9-69.9)	0.45 (0.25-0.84)	

Data from the Surveillance, Epidemiology and End Results Program, 2003 to 2009. NEC, neuroendocrine carcinoma; DSS, disease-specific survival; OS, overall survival; *n*, number of cases; HR, hazard ratio; NS, not significant; CI, confidence interval; TNM, tumor-lymph nodes-metastasis; LN, lymph node; ER, estrogen receptor; PR, progesterone receptor.

**Table 4:** Multivariate analysis of independent prognostic factors for DSS and OS in patients with NEC of the breast

**Group**	**DSS**		**OS**	
	**HR (95% CI)**	***P***	**HR (95% CI)**	***P***
Age (>60 years *vs* ≤60 years)	1.82 (0.86-3.85)	NS	2.02 (1.17-3.52)	0.012
Size (>20 mm *vs* ≤20 mm)	1.82 (0.91-3.64)	NS	1.44 (0.89-2.32)	NS
LN (positive *vs* negative)	2.54 (1.59-4.06)	<0.0001	2.05 (1.51-2.80)	<0.0001
Grade (III *vs* I, II)	1.18 (0.80-1.74)	NS	1.24 (0.95-1.62)	NS
ER status (positive *vs* negative)	1.23 (0.82-1.84)	NS	0.95 (0.69-1.32)	NS
PR status (positive *vs* negative)	0.59(0.41-0.86)	0.006	0.82 (0.60-1.10)	NS
Radiation (yes *vs* no)	0.79 (0.41-1.55)	NS	0.76 (0.47-1.22)	NS
Surgery (yes *vs* no)	0.39 (0.16-0.96)	0.041	0.57 (0.27-1.20)	NS

DSS, disease-specific survival; OS, overall survival; NEC; neuroendocrine carcinoma; HR, hazard ratio; CI, confidence interval; NS, not significant; LN, lymph node; ER, estrogen receptor; PR, progesterone receptor.

To determine whether neuroendocrine differentiation itself has prognostic significance, we pooled the NEC and IMC-NOS cases together and performed multivariate analyses based on all the known prognostic factors in addition to neuroendocrine differentiation. As shown in Table 5, neuroendocrine differentiation was an independent adverse prognostic factor for both OS and DSS (both *P* < 0.0001).

**Table 5:** Multivariate analysis of independent prognostic factors for DSS and OS in patients with invasive carcinoma of the breast (pooled NEC and IMC-NOS)

**Group**	**DSS**		**OS**	
	**HR (95% CI)**	***P***	**HR (95% CI)**	***P***
Age, years (>60 *vs* ≤60)	1.14 (1.13-1.16)	<0.0001	2.62 (2.60-2.65)	<0.0001
Tumor size, mm (>20 *vs* ≤20)	1.65 (1.64-1.66)	<0.0001	1.34 (1.33-1.35)	<0.0001
LN (positive *vs* negative)	1.98 (1.97-2.00)	<0.0001	1.64 (1.63-1.65)	<0.0001
Grade (III *vs* I, II)	1.26 (1.25-1.27)	<0.0001	1.12 (1.11-1.13)	<0.0001
ER status (positive *vs* negative)	0.82 (0.81-0.83)	<0.0001	0.90 (0.89-0.91)	<0.0001
PR status (positive *vs* negative)	0.88 (0.87-0.89)	<0.0001	0.93 (0.92-0.94)	<0.0001
Neuroendocrine (positive *vs* negative)	1.80 (1.36-2.37)	<0.0001	1.84 (1.50-2.26)	<0.0001
Radiation (yes *vs* no)	0.90 (0.89-0.91)	<0.0001	0.75 (0.74-0.76)	<0.0001
Surgery (yes *vs* no)	0.44 (0.43-0.45)	<0.0001	0.45 (0.46-0.47)	<0.0001

DSS, disease-specific survival; OS, overall survival; NEC; neuroendocrine carcinoma; IMC-NOS, invasive mammary carcinoma, not otherwise specified; HR, hazard ratio; CI, confidence interval; LN, lymph node; ER, estrogen receptor; PR, progesterone receptor.

### Clinical Significance of 2003 WHO Diagnostic Criteria for Mammary NEC

Mammary NEC has been a controversial entity. Variable clinical outcomes have been reported by different studies, partially due to inconsistent diagnostic criteria. In 2003, WHO implemented diagnostic criteria for this entity, requiring that >50% of the tumor cells express neuroendocrine markers.

**Figure 4:**
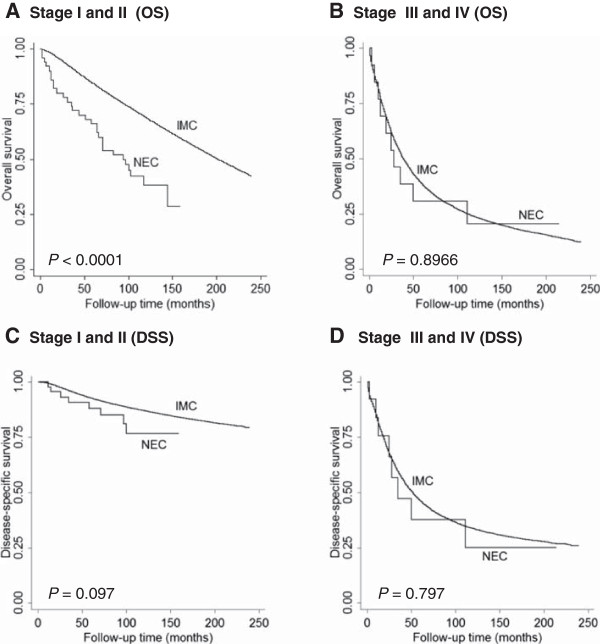
**Overall survival (OS) and disease-specific survival (DSS) comparisons between neuroendocrine carcinoma (NEC) and invasive mammary carcinoma, not otherwise specified (IMC) diagnosed between 1998 and 2002.** Although OS was significantly shorter in NEC than in IMC-NOS in early stage disease **(A)**, there was no difference in DSS between NEC and IMC-NOS **(C)**. There was no difference in both OS and DSS between NEC and IMC-NOS in advanced stage disease **(B and D)**.

We identified 72 additional mammary NEC based on the same ICD codes in the SEER database between 1998–2002, when the diagnostic criteria for mammary NEC were not uniformly applied. We performed survival analyses on those 72 cases, and showed no statistically significant difference in DSS for early stage (stage I-II) patients, and no difference in either OS or DSS in advanced stage (stage II-IV) patients (Figure 4). These results suggest that before 2003, some of the mammary NEC included in the SEER database may be those cases with focal NE differentiation (i.e., <50% of the tumor cells expressing neuroendocrine markers). As studies have shown that focal NE differentiation has no prognostic significance as compared with mammary carcinoma, NOS [5,14], our results from the SEER database between 1998–2002 further confirm the importance of applying 2003 diagnostic criteria for mammary NEC.

## Discussion

NEC of the breast is a rare disease. Only 6 case series have been reported in the literature, the largest comprising 74 cases [6-11]. With the 142 SEER cases reported here, this is the largest series reported to date and the first population study of mammary NEC.

The incidence of NEC of the breast has not been reported. Although NEC was estimated in 2003 to represent 2-5% of breast carcinomas [13], we found from our analysis of SEER data released in April 2012 that the incidence of mammary NEC is much lower. The age-adjusted incidence is 0.41 per 1 million-years in the female population of the U.S., and NEC comprises <0.1% of all mammary carcinomas. Despite the low incidence of male breast carcinomas overall, the SEER data showed that NEC was proportionally more common in men than IMC-NOS (2.1% of all NEC; 0.8% of all IMC-NOS).

Because mammary NEC has not been well studied, its clinicopathologic features and outcome are poorly characterized. Among the 6 reported series studies, 2 studies with 35 and 10 patients showed no difference in outcome from IMC-NOS [6,9], and 3 studies with 13, 12, and 7 patients showed better prognosis in NEC [7-10]. The present study, representing a substantially larger cohort, showed a much poorer clinical outcome for mammary NEC than for IMC-NOS. This result was consistent with our previous report of 74 NEC cases from a single institution [11]. In the present study, median survival duration of NEC cases was much shorter than that of IMC-NOS cases (26 months in NEC; 34 months in IMC-NOS).

Like our previous study, this population-based study showed that a majority of the NECs were ER and/or PR positive (68%), though the proportion of ER- and PR-positive cases was slightly lower than that previously reported. The present study also showed that NEC tended to occur in older patients (mean age 64 years) than IMC-NOS (mean 61 years-old) and to present at higher clinical stages with larger tumors (mean 32 mm compared to 23 mm in IMC-NOS) and more frequent regional lymph node metastasis. Although NEC was often associated with less favorable clinicopathologic features, multivariate analyses showed that only older age (>60 years) and positive lymph node status were independent prognostic factors for OS, and only positive lymph node status, negative PR status and lack of surgical treatment were independent prognostic factors for DSS. When we compared NEC with IMC-NOS at the same clinical stage, both OS and DSS were statistically shorter in NEC than in IMC-NOS. Interestingly, when we pooled all the mammary carcinoma together, including NEC, and analyzed independent prognostic factors using multivariate analysis, neuroendocrine differentiation was revealed as an adverse prognostic factor independent of other prognostic factors, including greater age, larger tumor size, and higher histologic grade.

## Conclusions

In summary, this population-based study showed that NEC is an aggressive mammary carcinoma subtype with significantly shorter OS and DSS than IMC-NOS. It tends to present at greater age, with larger tumor size, higher histologic grade, and higher clinical stage. NEC also tends to be ER/PR positive, but positive ER status does not appear to confer a prognostic benefit as it does in other invasive mammary carcinomas. As information regarding systemic treatment, including hormonal therapy and chemotherapy, was not available in the SEER database, we could not analyze whether such therapies would make a difference in outcome in this disease. Our multivariate analyses showed, however, that radiation therapy did not prolong survival of patients with mammary NEC.

## Competing interests

The authors have no financial disclosures or conflicts of interest.

## Authors’ contributions

JW and YW contributed to the study design, analysis, interpretation and manuscript preparation. BW, CTA, JH contributed to data interpretation and manuscript revision. SCA contributed data interpretation, manuscript preparation and revision. All authors read and approved the final manuscript.

## Pre-publication history

The pre-publication history for this paper can be accessed here:

http://www.biomedcentral.com/1471-2407/14/147/prepub

## References

[B1] Cubilla AL Woodruff JM Primary carcinoid tumor of the breast: a report of 8 patients Am J Surg Pathol 1977 1 283 292 10.1097/00000478-197712000-00001

[B2] Fisher ER Palekar AS Solid and mucinous varieties of so-called mammary carcinoid tumors Am J Clin Pathol 1979 72 6 909 916 229725 10.1093/ajcp/72.6.909

[B3] Azzopardi JG Muretto P Goddeeris P Eusebi V Lauweryns JM ‘Carcinoid’ tumours of the breast: the morphological spectrum of argyrophil carcinomas Histopathology 1982 6 5 549 569 10.1111/j.1365-2559.1982.tb02750.x 6183185

[B4] Papotti M Macri L Finzi G Capella C Eusebi V Bussolati G Neuroendocrine differentiation in carcinomas of the breast: a study of 51 cases Semin Diagn Pathol 1989 6 2 174 188 2503862

[B5] Miremadi A Pinder SE Lee AH Bell JA Paish EC Wencyk P Elston CW Nicholson RI Blamey RW Robertson JF Ellis IO Neuroendocrine differentiation and prognosis in breast adenocarcinoma Histopathology 2002 40 3 215 222 10.1046/j.1365-2559.2002.01336.x 11895486

[B6] Sapino A Righi L Cassoni P Papotti M Gugliotta P Bussolati G Expression of apocrine differentiation markers in neuroendocrine breast carcinomas of aged women Mod Pathol 2001 14 8 768 776 10.1038/modpathol.3880387 11504836

[B7] Zekioglu O Erhan Y Ciris M Bayramoglu H Neuroendocrine differentiated carcinomas of the breast: a distinct entity Breast 2003 12 4 251 257 10.1016/S0960-9776(03)00059-6 14659309

[B8] Rovera F Masciocchi P Coglitore A La Rosa S Dionigi G Marelli M Boni L Dionigi R Neuroendocrine carcinomas of the breast Int J Surg 2008 6 Suppl 1 S113 S115 19167937 10.1016/j.ijsu.2008.12.007

[B9] Makretsov N Gilks CB Coldman AJ Hayes M Huntsman D Tissue microarray analysis of neuroendocrine differentiation and its prognostic significance in breast cancer Hum Pathol 2003 34 10 1001 1008 10.1053/S0046-8177(03)00411-8 14608533

[B10] Lopez-Bonet E Alonso-Ruano M Barraza G Vazquez-Martin A Bernado L Menendez JA Solid neuroendocrine breast carcinomas: incidence, clinico-pathological features and immunohistochemical profiling Oncol Rep 2008 20 6 1369 1374 19020716

[B11] Wei B Ding T Xing Y Wei W Tian Z Tang F Abraham S Nayeemuddin K Hunt K Wu Y Invasive neuroendocrine carcinoma of the breast: a distinctive subtype of aggressive mammary carcinoma Cancer 2010 116 19 4463 4473 10.1002/cncr.25352 20572042

[B12] SEER SEER claims files 2012 http://seer.cancer.gov/data/

[B13] Tavassoli FA Devilee P Pathology and Genetics: Tumours of the Breast and Female Genital Organs. WHO Classification of Tumours series, Volume 4 2003 3 Lyon, France: IARC Press 32 34

[B14] van Krimpen C Elferink A Broodman CA Hop WC Pronk A Menke M The prognostic influence of neuroendocrinedifferentiation in breast cancer: results of a longterm follow-up study Breast 2004 13 329 333 10.1016/j.breast.2003.11.008 15325669

